# The ‘R sign’ as a potential risk factor for progression of uncomplicated Type B aortic dissection: a case series

**DOI:** 10.1093/ehjcr/ytag707

**Published:** 2026-09-18

**Authors:** Dan Rong, Shaofan Wang, Jianhan Yin, Yangyang Ge, Feng Liu, Wei Guo

**Affiliations:** Department of Vascular and Endovascular Surgery, the First Medical Centre, Chinese PLA General Hospital, 28 Fuxing Road, Beijing 100853, China; School of Medicine, Nankai University, 94 Weijin Road, Tianjin 300071, China; School of Medicine, Nankai University, 94 Weijin Road, Tianjin 300071, China; Department of Vascular and Endovascular Surgery, the First Medical Centre, Chinese PLA General Hospital, 28 Fuxing Road, Beijing 100853, China; Department of Vascular and Endovascular Surgery, the First Medical Centre, Chinese PLA General Hospital, 28 Fuxing Road, Beijing 100853, China; Department of Vascular and Endovascular Surgery, the First Medical Centre, Chinese PLA General Hospital, 28 Fuxing Road, Beijing 100853, China

**Keywords:** Aortic dissection, Radiological risk factor, R sign, Case report

## Abstract

**Background:**

Complicated and uncomplicated Type B aortic dissection (TBAD) need distinct therapeutic pathways. While TBAD is a complex and dynamic clinical entity, ∼75% of cases present as uncomplicated initially. However, up to half of these may progress to complicated TBAD, highlighting the need for better prognostic tools. In this case series, we report a novel potential radiological risk factor for progression, the ‘R sign’, to contribute to a deeper understanding of this disease’s behaviour.

**Case summary:**

We present two male patients with TBAD. Both cases exhibited a localized dissection at the top of the aortic arch’s greater curvature on computed tomography angiography (CTA), which we term the ‘R sign’. Both patients were initially admitted with uncomplicated TBAD. Patient 1: a 46-year-old man was stable on medical therapy until experiencing sudden chest tightness, at which point CTA confirmed progression to Type A dissection. Patient 2: a 54-year-old man, also managed medically for uncomplicated TBAD, suffered a sudden recurrence of back pain, with CTA confirming progression to Type A dissection.

**Conclusion:**

This case series highlights a previously unrecognized subtype of aortic dissection. In both presented cases, this specific form of TBAD progressed to Type A during the acute phase. Early identification of this high-risk uncomplicated TBAD variant is critical for patient prognosis, as it may need urgent intervention as a complicated TBAD. We present this entity to raise awareness and call for further multi-centre studies to validate our observations in larger cohorts.

Learning points

- The possibility of progression should be considered in uncomplicated aortic dissection with the imaging feature of the ‘R sign’.
- The R sign can be easily overlooked on axial views, and evaluation using tangential views or volume rendering is recommended.

## Introduction

Acute Type B aortic dissection (TBAD) represents a critical cardiovascular emergency with a variable clinical trajectory. Current management strategies are fundamentally guided by the binary classification of TBAD at presentation into complicated and uncomplicated forms.^1,2^ Initially, up to 75% of TBAD cases are classified as uncomplicated TBAD and managed conservatively.^3,4^ However, this binary classification is increasingly recognized as insufficient, as up to half of patients initially presenting with uncomplicated TBAD experience progression to complicated ones.^4^ This highlights the existence of a high-risk uncomplicated TBAD phenotype that is poorly captured by current classification schemes. Consequently, there is a pressing clinical need to identify reliable imaging and morphological risk factors that can predict progression at the earliest stage.

In this report, we introduce a novel radiographic finding, referred to as the ‘R sign’, characterized by a localized dissection at the apex of the aortic arch’s greater curvature. We present two patients with initially uncomplicated TBAD who exhibited this sign, both of whom rapidly progressed to Type A dissection during the acute phase. This suggests that the ‘R sign’ may represent a distinct, high-risk subtype of uncomplicated TBAD. The goal of this study is to highlight this potential risk factor as a basis for future validation and improve prognostic stratification.

## Summary figure

**Figure:**
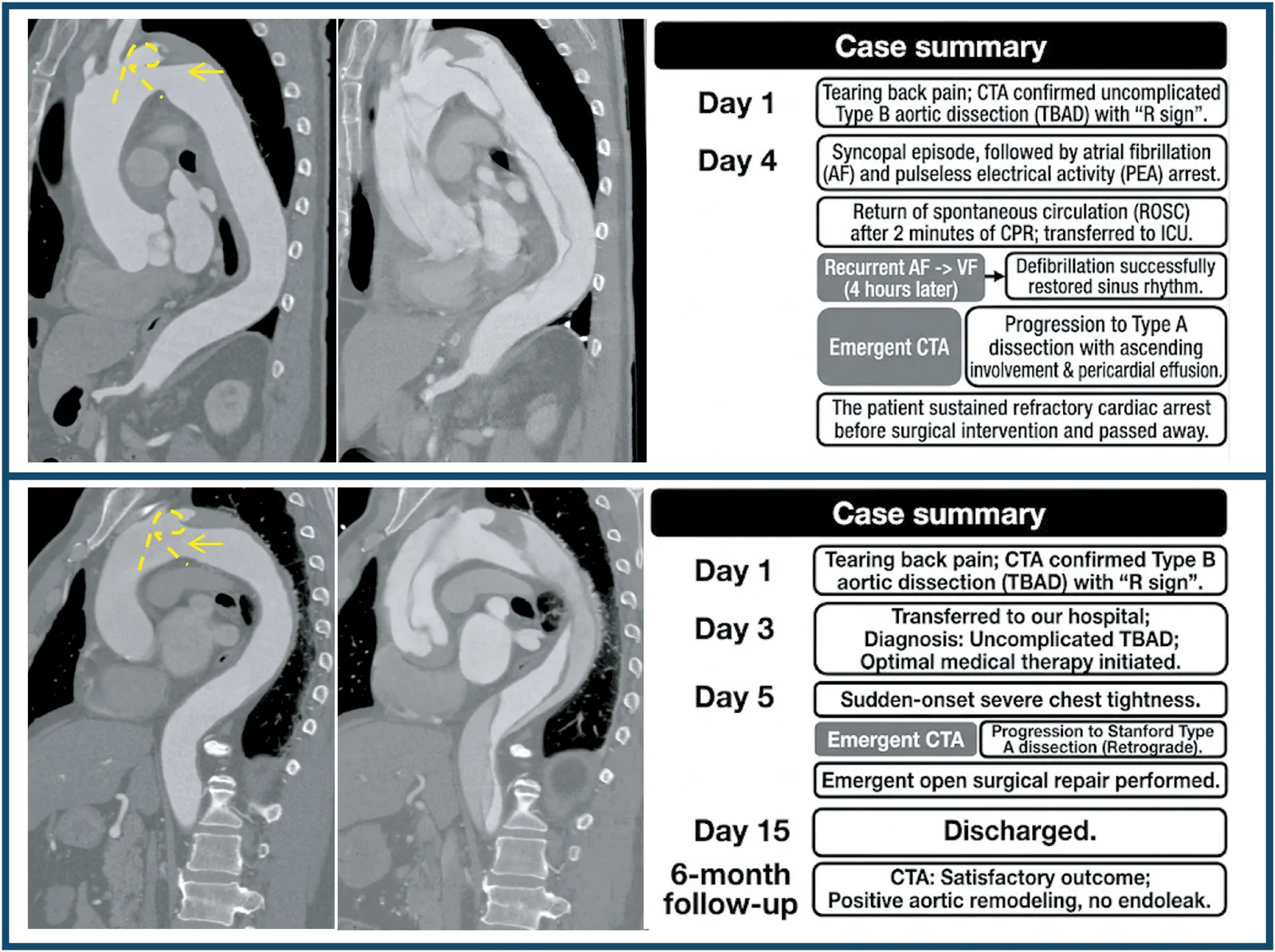


## Case 1

A 46-year-old male presented to the emergency department following transfer from a local hospital, 2 days after the onset of sudden, severe tearing back pain. He had a history of hypertension. He had no personal history suggestive of connective tissue disorders nor any positive family history of connective tissue disease. On admission, his blood pressure was 160/100 mmHg with a heart rate of 85 b.p.m. Physical examination revealed no pulse deficits or signs of organ malperfusion. An electrocardiogram showed sinus rhythm without ischaemic changes.

Emergency computed tomography angiography (CTA) confirmed a TBAD, with the primary entry tear located distal to the left subclavian artery. A critical imaging finding was noted: a localized dissection at the apex of the greater curvature of the aortic arch, extending 38 mm in length. This segment featured a blind-ended false lumen with a maximal diameter of 10 mm and no distal re-entry tear. We define this specific morphology as the ‘R sign’ (*Figure 1A* and *B*). The maximum aortic diameter at the dissection site was 26 mm. In the absence of complications, a diagnosis of uncomplicated TBAD was made, and optimal medical therapy was initiated.

**Figure 1:**
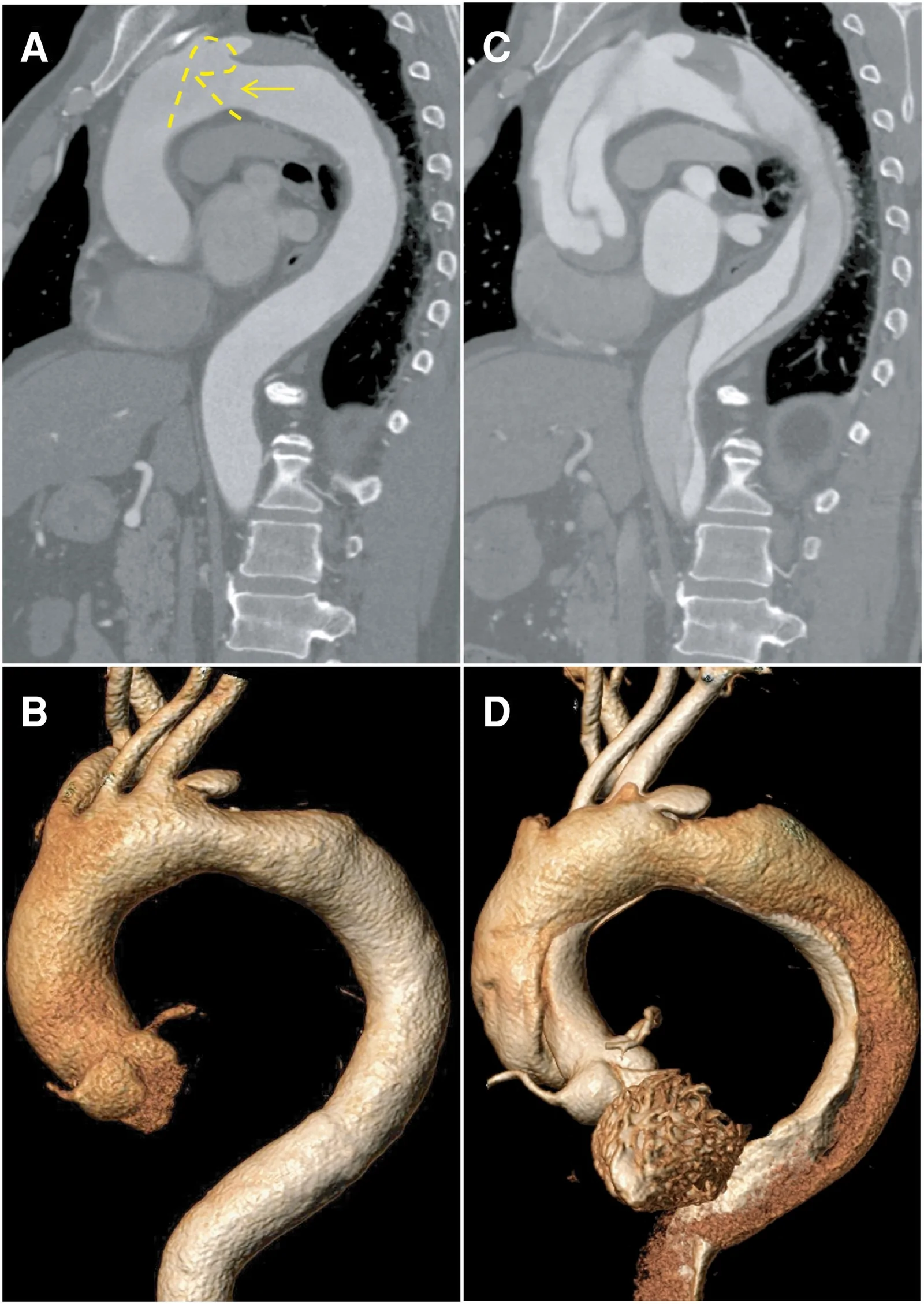
Computed tomography angiography of the aorta in Patient 1 revealed a localized blind-ending false lumen at the apex of the aortic arch upon admission, with no complicated features, characterized by the ‘R sign’ on imaging (*A* and *B*). Following 3 days of conservative management with best medical therapy, the condition progressed to a Type A aortic dissection (*C* and *D*).

The patient’s pain had resolved on admission. However, on hospital Day 3, he developed sudden-onset severe chest tightness. An emergent repeat CTA demonstrated progression to a Stanford Type A aortic dissection, with retrograde extension of the dissection flap into the ascending aorta (*Figure 1C*).

The patient underwent emergent open surgical repair with ascending aorta and total arch replacement using a frozen elephant trunk procedure. The postoperative course was uneventful, and he was discharged on postoperative Day 10. A follow-up CTA at 6 months confirmed a satisfactory surgical outcome, with no endoleak and positive aortic remodelling.

Computational fluid dynamics (CFD) analysis revealed that, in this aortic dissection case, the mean pressure within the blind-ended false lumen was higher than that in the true lumen, suggesting a relatively isolated haemodynamic environment with limited effective communication between the false and true lumens (*Figure 2A*). Furthermore, the false lumen exhibited markedly elevated relative residence time (RRT), extremely low time-averaged wall shear stress (TAWSS), and a high oscillatory shear index (OSI) near its proximal entry site (*Figure 2B–D*).

**Figure 2:**
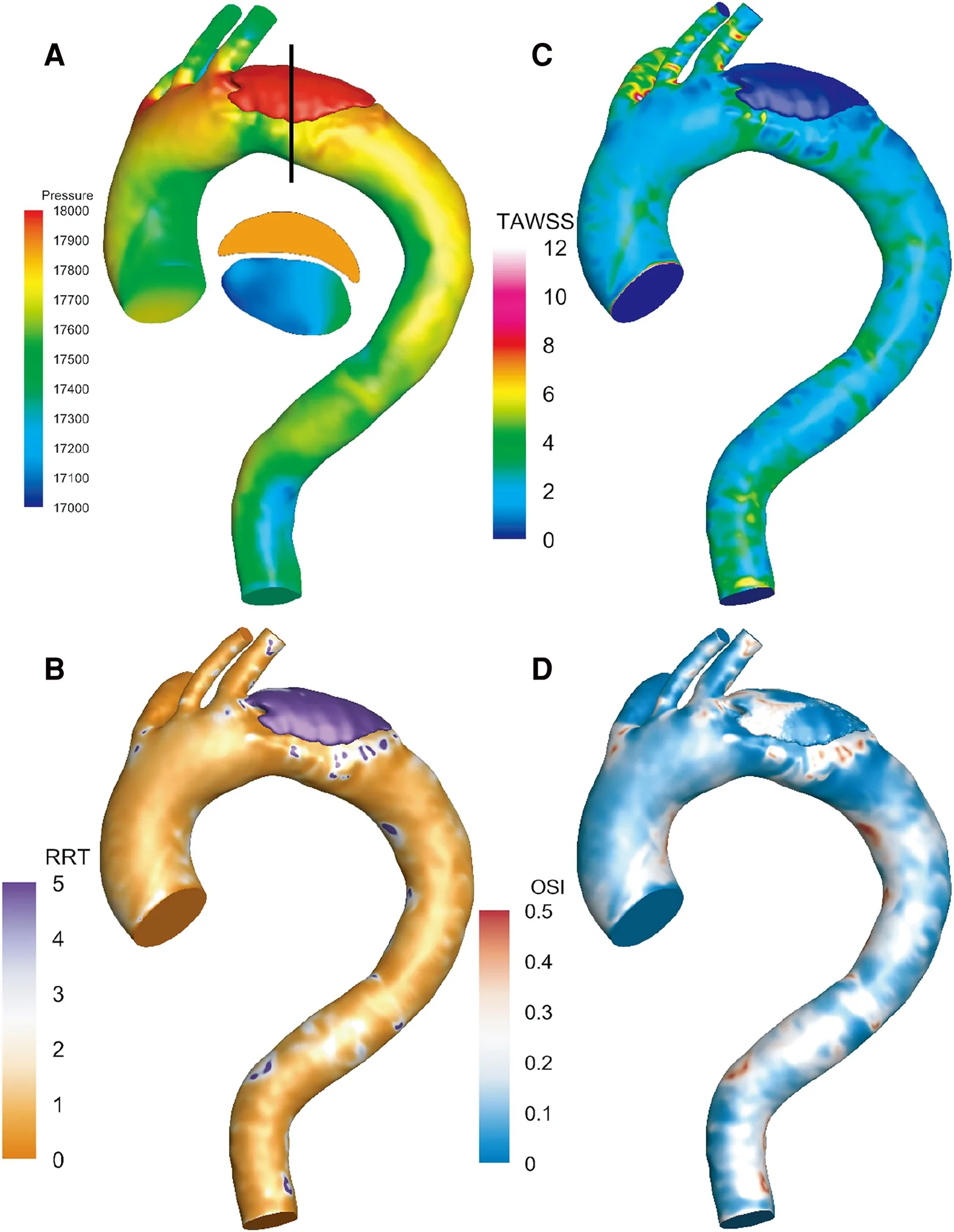
Computational fluid dynamics results of aortic computed tomography angiography upon admission in Patient 1. The mean pressure in the false lumen was significantly higher than that in the true lumen (*A*). Meanwhile, TAWSS, RRT, and OSI showed consistent trends, all being significantly lower in the false lumen compared to the true lumen at the same central line plane (*B–D*). TAWSS, time-averaged wall shear stress; RRT, relative residence time; OSI, oscillatory shear index.

## Case 2

A 54-year-old male presented with 8 h of acute, tearing back pain. His medical history included hypertension, hyperlipidaemia, and a significant 30-pack-year smoking history. She also had no personal history suggestive of connective tissue disorders or any positive family history of connective tissue disease. Initial evaluation revealed a blood pressure of 150/95 mmHg and a heart rate of 95 b.p.m. Computed tomography angiography confirmed an uncomplicated TBAD, but it also revealed the characteristic ‘R sign’—a localized, blind-ended false lumen at the apex of the aortic arch, measuring 45 mm in length and 12 mm in maximal diameter (*Figure 3A* and *B*). Optimal medical therapy was commenced.

**Figure 3:**
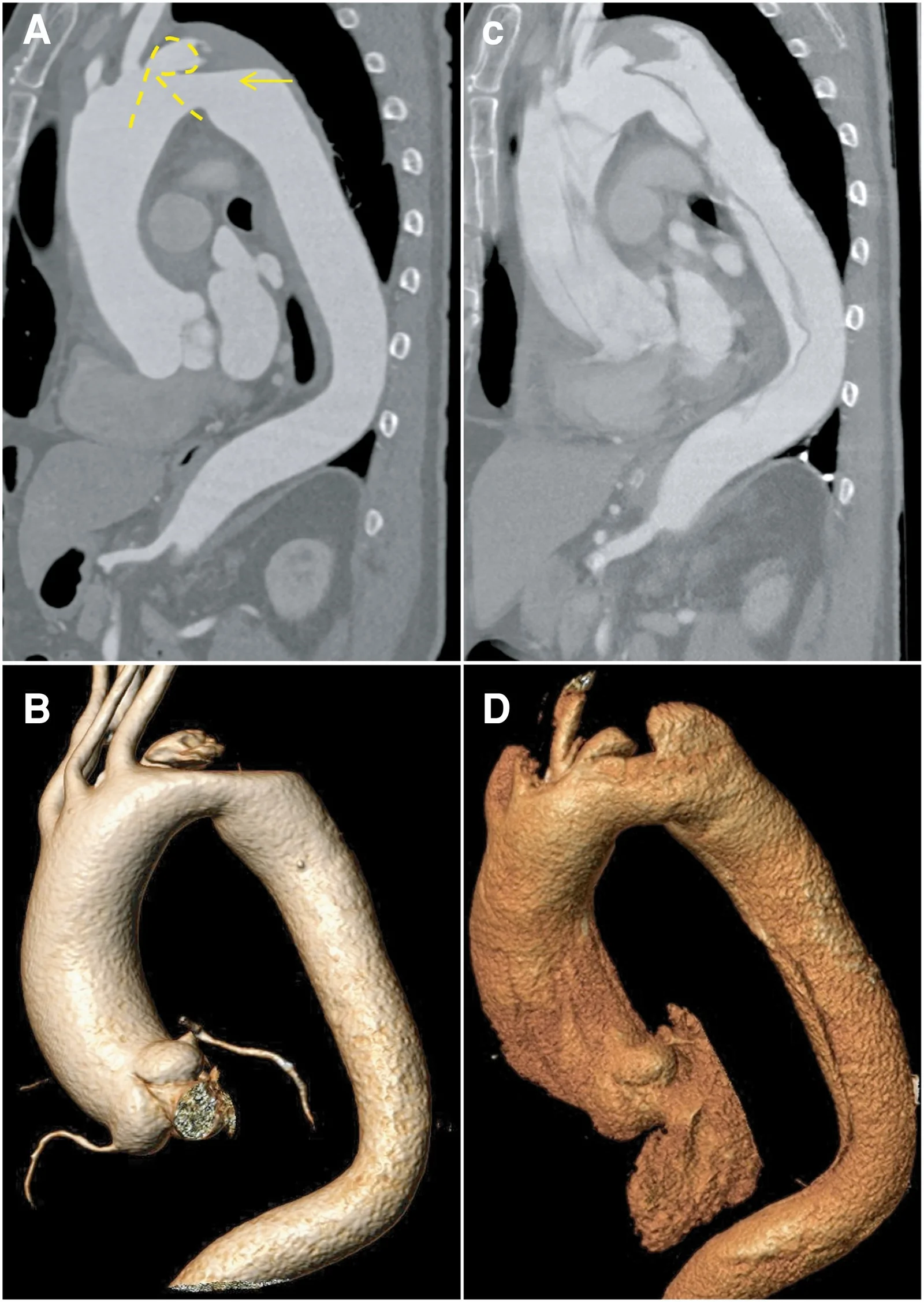
Patient 2 presented with a similar localized false lumen at the apex of the aortic arch upon admission, without ischaemia of organs or branch vessels (*A* and *B*). On the fourth day of conservative treatment after admission, the patient also progressed to a Type A aortic dissection (*C* and *D*).

On hospital Day 4, during conservative management, the patient’s condition deteriorated suddenly. He experienced a syncopal episode, followed by atrial fibrillation and subsequent pulseless electrical activity cardiac arrest. Return of spontaneous circulation was achieved after 2 min of cardiopulmonary resuscitation, and he was transferred to the intensive care unit. Four hours later, he developed recurrent atrial fibrillation, which rapidly degenerated into ventricular fibrillation. Defibrillation was performed twice at 200 J, successfully restoring sinus rhythm.

An emergency CTA confirmed progression to a Type A aortic dissection with involvement of the ascending aorta and pericardial effusion (*Figure 3C*). A multidisciplinary heart team considered emergency surgery, but the patient sustained a final, refractory cardiac arrest before any intervention could be performed. Despite prolonged advanced life support, return of spontaneous circulation could not be achieved, and the patient passed away. Post-mortem histopathological examination and molecular genetic testing were not performed, as the patient’s family did not consent to an autopsy.

Computational fluid dynamics analysis of this case demonstrated a similar haemodynamic profile to that observed in Patient 1. The blind-ended false lumen similarly displayed an elevated mean pressure compared to the true lumen (*Figure 4A*). Flow analysis further identified a region of profoundly disturbed haemodynamics, characterized by a significantly prolonged RRT, exceedingly low TAWSS, and a high OSI localized at the proximal segment of the false lumen (*Figure 4B–D*). These consistent findings reinforce the hypothesis that the ‘R sign’ morphology creates a distinct, high-risk haemodynamic microenvironment.

**Figure 4:**
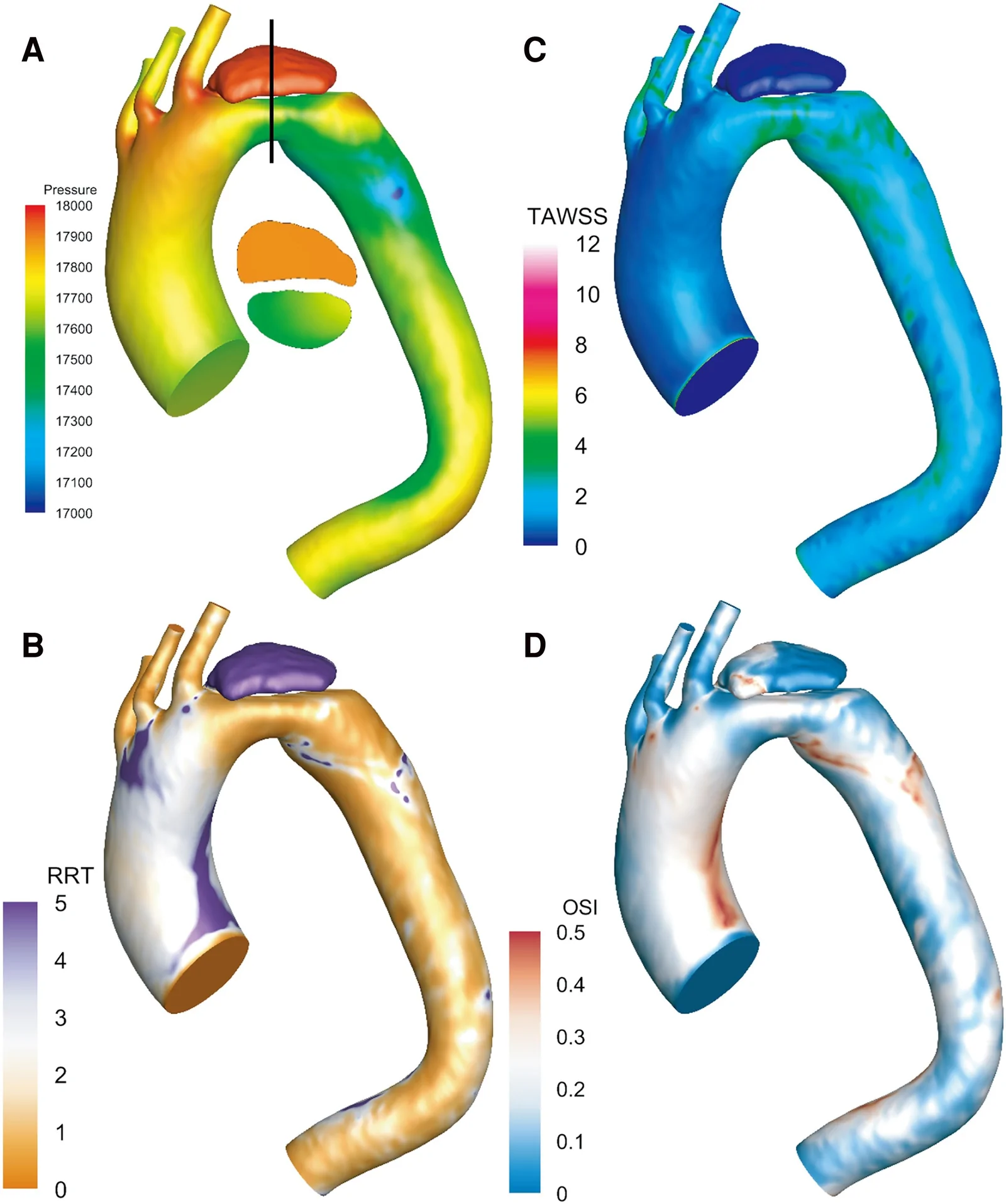
Computational fluid dynamics results of aortic CTA at admission for Patient 2, presenting similarly with a higher mean pressure in the false lumen (*A*) and lower TAWSS, RRT, and OSI compared to the true lumen (*B–D*). TAWSS, time-averaged wall shear stress; RRT, relative residence time; OSI, oscillatory shear index.

## Discussion

The present case series describes two patients with initially uncomplicated acute TBAD who shared a distinct radiographic morphology, the ‘R sign’, defined as a localized, blind-ended false lumen at the apex of the aortic arch’s greater curvature. Although genetic testing was not performed, thorough history and physical examination did not suggest heritable connective tissue disease. Both patients experienced rapid retrograde extension into the ascending aorta during the acute phase. This consistent clinical trajectory, together with the haemodynamic characteristics demonstrated by CFD, suggests that the ‘R sign’ may represent a previously unrecognized high-risk phenotype within the spectrum of uncomplicated TBAD.

Current European Society of Cardiology guidelines emphasize the dichotomous classification of TBAD into uncomplicated and complicated forms, recommending optimal medical therapy for the former and early intervention for the latter.^5^ However, the guidelines acknowledge that a subset of patients initially categorized as ‘uncomplicated’ may subsequently deteriorate, and they highlight the importance of early imaging predictors to identify high-risk patients. Existing morphological predictors, such as large aortic diameter, partial thrombosis, and large entry tears, have been associated with adverse events, yet none fully explain the rapid progression described in our patients.^6–8^

The ‘R sign’ demonstrated several features that may explain its clinical instability. First, its blind-ended configuration created an isolated false-lumen cavity without distal re-entry, a geometry that CFD analysis confirmed to be haemodynamically unfavourable. Both cases exhibited elevated false lumen pressure relative to the true lumen, suggesting a trapped pressurized lumen prone to progression. Moreover, the region demonstrated extremely low TAWSS, high OSI, and significantly prolonged RRT, all of which have been linked to disturbed flow, impaired endothelial integrity, and heightened mechanical stress on the aortic wall.^9,10^ These unique haemodynamic patterns may explain the predisposition for rapid false-lumen expansion and retrograde dissection into the ascending aorta.

Importantly, this pattern differs from the classical entry-tear-related progression typically observed in TBAD. Rather than originating from a large primary tear, the ‘R sign’ appears to represent a secondary localized instability at the greater curvature of the aortic arch, which is characterized by high curvature and complex flow even under physiological conditions. The reproducibility of this morphology and haemodynamic profile across both patients strengthens the hypothesis of a distinct risk phenotype.

The management implications of these findings are significant. Under current guideline-based management, both patients met criteria for uncomplicated TBAD at presentation and therefore received optimal medical therapy (OMT) with close surveillance. However, the rapid deterioration in both cases raises the question of whether patients exhibiting the ‘R sign’ should be considered for more aggressive early intervention even in the absence of classical complications. Prophylactic thoracic endovascular aortic repair (TEVAR) in selected high-risk uncomplicated TBAD patients is already supported by emerging evidence.^11–13^ Therefore, identifying reliable imaging markers for risk stratification is critical. The ‘R sign’ may serve as one such marker, pending further validation.

## Conclusion

In conclusion, the ‘R sign’ represents a novel imaging feature associated with early progression to Type A aortic dissection in seemingly uncomplicated TBAD. The combination of focal anatomical instability and abnormal haemodynamic forces provides a plausible mechanistic explanation for this behaviour. Recognition of this morphology may improve early risk stratification and guide more individualized management strategies. Further multi-centre validation is warranted to determine whether the ‘R sign’ should be incorporated into future TBAD classification and management algorithms.

## Data Availability

The data underlying this article will be shared on reasonable request to the corresponding author.

## Lead author biography

Dr Dan Rong is a vascular surgeon who completed his residency training at Chinese PLA General Hospital and is currently undertaking fellowship training. His main research interests focus on the treatment of aortic diseases and the development of endovascular aortic devices. His clinical interests include aortic dissection, aortic aneurysm, and peripheral artery disease.
